# Comparative analysis of the nutritional composition, digestibility, metabolomics profiles and growth influence of cow, goat and sheep milk powder diets in rat models

**DOI:** 10.3389/fnut.2024.1428938

**Published:** 2024-11-22

**Authors:** Chun Yang, Jiancun Pan, Shaojie Pang, Shuang Hu, Miao Liu, Xinyan Zhang, Liping Song, Xiangnan Ren, Zhongli Wang

**Affiliations:** ^1^School of Public Health, Capital Medical University, Beijing, China; ^2^Feihe Research Institute,Heilongjiang Feihe Dairy Co., Ltd., Beijing, China; ^3^Children’s Hospital of Nanjing Medical University, Nanjing, China; ^4^National Institute for Nutrition and Health, Chinese Center for Disease Control and Prevention, Beijing, China; ^5^Department of Rehabilitation Medicine, The Second Affiliated Hospital of Jiaxing University, The Second Hospital of Jiaxing, Jiaxing, China; ^6^School of Medicine and Nursing, Huzhou University, Huzhou, Zhejiang, China

**Keywords:** nutritional composition, growth performance, digestibility, metabolomics profiles, cow milk, goat milk, sheep milk, rat models

## Abstract

**Introduction:**

The diversity of dairy products and the increasing consumption levels have led to a growing interest in goat and sheep milk, which are rich in essential nutrients and functional components. The study aims to explore the nutritional composition, growth performance, digestibility, and serum metabolic differences of milk powders from cow, goat, and sheep using LC–MS/MS-based metabolomics in rat models.

**Methods:**

Sixty male Sprague-Dawley rats were fed with whole cow, goat, and sheep milk powder samples , and their feces and urine were analyzed for fat and protein content. LC/MS analysis was conducted using a Dionex UltiMate 3000 UHPLC system coupled with a Thermo Q EXACTIVE mass spectrometer, with data processed using Wekemo Bioincloud for quality control, normalization, comparisons with the KEGG database, statistical analyses, and selection of differential metabolites.

**Results:**

The sheep milk powder showed highest protein and fat content level, while cow and goat milk powders separately demonstrated higher lactose and carbohydrate levels. Each milk powder had a unique mineral profile, with sheep milk powder containing the highest calcium content. All groups exhibited consistent growth in body weight and high rates of protein and fat digestibility. Metabolomics analysis revealed distinct metabolic profiles, with goat milk powder linked to steroid hormone biosynthesis and sheep milk powder associated with hormone regulation and bile acid pathways.

**Conclusion:**

This study offers valuable insights into the metabolic implications of different milk powder sources, informing dietary choices and facilitating the development of targeted public health strategies to optimize nutritional intake and promote overall well-being.

## Introduction

People’s choice of dairy products has become more diverse with changes in consumption levels. Compared with the year 2011 level, dairy consumption is expected to increase by 58% by 2050 (1). Therefore, the scale of the dairy industry and different types of dairy product will gradually expand. Goat milk production represents 2.1% of global milk production (2). Additionally, 95% of the world’s goat population is located in Asia, Africa, and Latin America. It is worth noting that Asia accounts for approximately 60% of the total goat population. China’s goat milk production increased from 54,000 tons in 1969 to 223,134 tons in 2018, with an impressive yearly average growth rate of 3.31% (3). Shanxi Province in China is home to the largest storage facility for goat milk (4). Goat milk contains relatively less lactose and fat but is rich in calcium, antimicrobial factors, antioxidants, and other functional components essential to human health (5). Similar to human breast milk, goat milk contains a high level of antimicrobial enzymes, such as lysozyme, which may enhance infants’ immunity against numerous infections (6). Due to its similarities with human breast milk, goat milk has been recognized as an important substitute for human breast milk (7). Sheep milk and products made from sheep milk have gained popularity in many populations and areas, especially among people with cow milk allergies. In ancient times, milk was primarily collected from small farms with a limited number of animals and consumed locally. However, with increasing urbanization and demand for dairy products in the early 1900s, the dairy industry underwent industrialization in more developed countries (8). Comparatively, sheep milk production in many countries still occurs on a small scale and is considered less significant in terms of volume. Nevertheless, sheep and goat milk have likely been used by humans for a longer time than cow milk, as sheep and goats were domesticated earlier than cattle (9).

Metabolomics has been employed in the investigation of dairy products through using an untargeted metabolomics approach by a gas chromatography coupled with mass spectrometry (GC–MS) to study the metabolite profiles of sheep’s and goat’s milk (10). Milk from cow, sheep, and goat in the ‘Alto Casertano’ region of Italy exhibits varying protein and amino acid profiles, with sheep milk having the highest total protein content level and goat milk containing unique amino acids such as taurine (10). In previous studies, researchers have utilized non-targeted Liquid Chromatography Mass Spectrometry (LC/MS) metabolomics approaches to characterize the profile of milk metabolites and identify differences between human milk and milk from cows, horses, and goats (11). However, those existing studies on dairy product metabolomics primarily focused on analyzing initial materials, lacking insights into *in vivo* metabolism. Thus, the current study aims to bridge this gap by providing a comprehensive understanding of the nutritional composition, growth performance, digestibility, and serum metabolic differences by using LC–MS/MS-based metabolomics analysis of rat models on milk powder diets from cow, goat, and sheep in order to explore potential metabolic pathways.

## Materials and methods

### Milk powder

The initial powdered materials obtained from the milk include full-cream cow milk powder sourced from New Zealand and the Netherlands, full-cream goat milk powder from New Zealand, the Netherlands, and Spain, and full-cream sheep milk powder from New Zealand, Italy, Spain, and Romania. These materials undergo a series of processes including filtration, purification, and sterilization to eliminate microorganisms, concentration to enhance richness, and spray drying to transform the liquid into a powder form.

### Testing of the nutritional components of milk powder samples

Sample was weighed 100 mg of milk powder samples and added them to 0.6 mL of water. After 15 min of sonication, 15 rounds of 30-s sonication and 1 min of off-sonication were performed in a Bioruptor sonication device (Diagenode) for homogenization, before proceeding with the nutritional component analysis. The basic nutrients were determined in accordance with the Chinese National Standard such as protein by Kjeldahl method (GB 5009.5–2016), fat by Gas chromatography method (GB 5009.168–2016), lactose by High Performance Liquid chromatography (HPLC) method (GB 5413.5–2010), moisture by direct drying method (GB 5009.3–2010), docosahexaenoic acid (DHA), linoleic acid, linolenic acid, and other fatty acids by acetyl chloride-methanol methyl esterification method (GB 5413.27–2010),amino acids by the Amino acid automatic analyzer (A300, Amino acid Analyzer, MembraPure GmbH) (GB 5413.27–2010), calcium, iron, zinc, sodium, potassium, magnesium, copper, manganese by atomic absorption method (GB 5413.21–2010), vitamin A by HPLC method (GB 5009.82–2016),vitamin B1 by HPLC method (GB 5009.84–2016), vitamin B2 by HPLC method (GB 5009.85–2016), and folic acid by HPLC method (GB 5413.16–2010).

### The formulation of milk powder feed

To equalize the protein levels among the three types of initial milk powder, whey protein powder corresponding to each type was used for supplementation based on the measured protein content in each type of initial milk powder. The milk powder solution was prepared at a concentration of 40% (wet basis), indicating that the milk powder comprised 40% of the total solution weight. Subsequently, the fat content in the milk powder and the added whey protein powder were adjusted to a consistent level by using full-cream cow milk powder from New Zealand and the Netherlands, full-cream goat milk powder from New Zealand, the Netherlands, and Spain, and full-cream sheep milk powder from New Zealand, Italy, Spain, and Romania, respectively, in the three types of milk powder solutions, the powder formulation should be presented in Supplementary Table S1. The solutions were then spray-dried to obtain powdered forms, which were subsequently physically compressed into rod-shaped forms (Nantong Teluofei Feed Technology Co., Ltd., Nantong, China). The resulting feeds underwent sterilization by using Co60 radiation at a dosage of 25 kGy.

### Animals and rearing conditions

The study has been approved by the Animal Ethics Committee of China Center for Disease Control and Prevention Ethics Committee (EAWE-2018-015), ensuring compliance with ethical guidelines for animal research. Sixty Sprague–Dawley rats (3–4 weeks old; male) were purchased from the Vital River Laboratory Animal Technology Co. Ltd. (Beijing, China). The rats were housed in individual metabolic cages. They were housed in an animal room maintained under standard conditions. The rats were provided with a maintenance feed and water *ad libitum*. The room conditions included a 12-h/12-h light/dark cycle, controlled temperature at 22°C, and access restrictions. The animals were kept under specific pathogen-free conditions, with daily temperature fluctuations being around 3°C, relative humidity ranging from 40 to 70%, airflow velocity of approximately 0.18 m/s, and a room air pressure gradient of 20–50 Pa. After 3 days of acclimation, animals were randomly divided into three groups, with 20 rats for each based on their body weights. Group names are as follows: C: whole cow milk group; G: whole goat milk group; S: whole sheep milk group. After 1 week of dietary feeding, each group was provided with the corresponding milk powder feed and *ad libitum* access to water, and the mice were anesthetized with 10% chloral hydrate (0.1 mL/10 g body weight). Blood samples were collected into EDTA-containing tubes by being drawn from the abdominal aorta, and were then centrifuged at 1,600 *g* for 20 min at 4°C. Collected plasma was aliquoted and snap-frozen by using liquid nitrogen.

### Collection and processing of fecal and urine samples in rat digestibility study

On the 4th day, a 24-h fasting period was commenced, with free access for rats to water. This was followed by a digestive experimental phase involving feeding from the morning of the 5th day until the morning of the 10th day. On the morning of the 5th day, the body weight of the rats was measured (in grams), and approximately 14 g of feed was provided. Feces and urine samples were collected in the morning and afternoon. From the morning of the 6th day to the morning of the 9th day, the weight of 5 mL centrifuge tubes (in grams), rat body weight (in grams), remaining feed weight (in grams), and the amount of feed replenished (approximately 14 g) were recorded to accurately measure the weight of feces and urine. Feces and urine samples were collected in the morning and afternoon of each day. On the morning of the 10th day, a 24-h period of fasting with unrestricted water access was implemented. Feces and urine were collected in the morning and afternoon of the 10th day, as well as in the morning of the 11th day. The urine samples were collected in centrifuge tubes and fixed with 10% hydrochloric acid, and feces were collected in 5 mL centrifuge tubes and fixed with 10% hydrochloric acid.

### Detection of metabolites related to fat and protein metabolism

The determination of fat content was carried out according to the method specified in GB5009.6–2016 “Determination of Fat in Foods.” Approximately 0.6 g of thoroughly mixed freeze-dried fecal samples and 10 g of urine samples were weighed. The fecal samples were then placed in a steam dish with approximately 20 g of quartz sand, and dried in an oven at 100 ± 5°C for 30 min. The dried samples were then finely ground and transferred into a filter paper tube. Following this, the filter paper tube was placed in the Soxhlet extractor’s extraction tube, connected to a dried receiving bottle, and subjected to extraction with petroleum ether for approximately 8 h. After the extraction was completed, the remaining solvent in the receiving bottle was evaporated on a water bath, dried in an oven at 100 ± 5°C for 1 h, and weighed until a constant weight was achieved.

Protein content was determined according to the method specified in GB5009.5–2016 “Determination of Protein in Foods.” Approximately 0.6 g of thoroughly mixed freeze-dried fecal samples and 10 g of urine (centrifuged at 6000 r/min) were weighed and transferred into a dried 100 mL Kjeldahl flask. Subsequently, 0.4 g of copper sulfate, 6 g of potassium sulfate, and 20 mL of sulfuric acid were added to the flask. The mixture was then heated until carbonization was complete, followed by the addition of water and subsequent transferred into a 100 mL volumetric flask for further processing. The process included the preparation of reagent blank tests, setting up of the Kjeldahl distillation apparatus, and subsequent titration of the distillate with standard sulfuric acid solution until the endpoint color turned to gray-blue.

The combined daily volume of urine samples from each animal group was recorded. A certain volume of urine was aliquoted for protein and fat content determination, and the total protein and total fat content in the urine were calculated. The combined feces from each animal group were dried daily, weighed, and the total weight was recorded. A portion of the feces was aliquoted for protein and fat content determination, and the total protein and total fat content in the feces were calculated. The apparent protein/fat digestibility rate was calculated as (food nitrogen/fat – fecal nitrogen/fat) divided by food nitrogen/fat, multiplied by 100%. The true protein/fat digestibility rate was calculated as [food nitrogen/fat – (fecal nitrogen/fat – metabolic fecal nitrogen/fat)] divided by food nitrogen/fat, multiplied by 100%. The net protein utilization rate was calculated as [food nitrogen – (fecal nitrogen – fecal metabolic nitrogen) – (urinary nitrogen – urinary metabolic nitrogen)] divided by food nitrogen, multiplied by 100%.

The biological value of proteins was calculated as [food nitrogen – (fecal nitrogen – nitrogen from fecal metabolism) – (urine nitrogen – endogenous urinary nitrogen)] divided by [food nitrogen – (fecal nitrogen – nitrogen from fecal metabolism)] multiplied by 100%. The net protein utilization rate (NPU), defined as the product of biological value and true digestibility rate, represented the efficiency of protein utilization in the body. Where fecal nitrogen and fecal fat were the nitrogen and fat content in the feces of rats from the group fed with nitrogen-free feed, a nitrogen-free diet formulation has been provided in Supplementary Table S2.

### Pretreatment

Serum samples were prepared by mixing 20 μL of serum with 200 μL of protein precipitant methanol-acetonitrile (V: V = 1:1) thoroughly, centrifuged at 10,000 rpm for 10 min, and 5 μL of serum were taken for LC–MS/MS analysis.

### Liquid chromatography-mass spectrometry analysis (LC/MS)

All LC/MS experiments were carried out by using a Dionex UltiMate 3,000 UHPLC (Thermo Scientific, MA) system equipped with a binary solvent delivery manager and a sample manager, coupled with a Thermo Q EXACTIVE operating in positive (ESI+) and negative (ESI−) electrospray ionization mode (Thermo Fisher Scientific, Sunnyvale, CA, United States). The liquid chromatography was conducted by using an Acquity BEH C18 column (50 mm × 2.1 mm, 1.7 μm particle size; Waters, Milford, MA, United States). Separation was achieved with solvent A [2 mmoL/L ammonium formate and 0.1% (v/v) formic acid] and solvent B (acetonitrile) with the following gradient at a cow rate of 0.25 mL/min: 0 min (5% B), 0–1 min (5% B), 1–5 min (5–60% B), 5–8 min (60–100% B), 8–11 min (100% B), 11–14 min (100–60% B), 14–15 min (60–5% B), and 15–18 min (5% B). The injection volume was 5 μL and the analysis time was 18 min. The mass spectrometric data were collected by using a Thermo Q EXACTIVE mass spectrometer. The mass spectrometer parameters in this experiment were as follows: the voltage was 2.8 kV; the sheath gas: 35 Arb; the auxiliary gas: 10 Arb; the capillary temperature: 350°C; the s-lens RF: 50. The resolution of the first full scan (Full scan) was 70,000; AGC target: 1e6; Maximum TT: 100 ms; the scan range: 70–1,050 m/z. The resolution of the secondary data dependency scan (Full MS/dd-MS2) was 35,000; AGC target: 1e5; Maximum TT:50 ms; NCE:20, 40, 60.

### Quality control

To ensure the consistency of the analysis process, it was reported that the quality control (QC) sample was initially prepared using 10 μL of serum from each sample. It was also noted that during the analysis, water samples and QC samples were injected once for every 10 samples to monitor sample preparation and instrument stability.

### Data processing

Raw data analysis was performed by using Wekemo Bioincloud, a free online platform for data analysis^1^. Raw data was processed using Proteowizard software (v3.0.8789) for quality assessment and quality control to detect outliers and remove metabolites or samples that exceeded threefold standard error during data preprocessing. Metabolomics workflow was based on the R (v3.1.3) language MetaboAnalystR package (12). Each metabolite was then compared with the KEGG database br08001 (13) to determine its percentage content in each biological role. Statistical analyses, including compound summaries and assessments of structural differences, were performed. Chemometric analysis of serum metabolites was conducted for all groups, utilizing principal component analysis (PCA), Partial Least Squares Discriminant Analysis (PLS-DA) (14), and Orthogonal Partial Least Squares Discriminant Analysis (OPLS-DA) (15). Differential serum metabolites between the groups were identified based on the variable importance in projection (VIP > 1) from OPLS-DA and the false discovery rate (FDR) from t-tests (FDR < 0.05). For the selection of differential metabolites, machine learning techniques such as random forest were employed, and only the results with FDR < 0.05 were shown. To provide intuitive insights into relationships, functional patterns, and metabolic pathways, correlation analyses, over-representation analysis (ORA) of pathways (15), topology analysis of pathways, and metabolic pathway maps, along with the topological structure of metabolites within the enriched pathways with pathway impact >0.1 and FDR < 0.05, were deemed key differential pathways between groups.

### Statistical analysis

The data was presented as the mean + standard deviation (SD) and was analyzed by one-way analysis of variance (one-way ANOVA). If significant (*p* < 0.05) differences were found by the ANOVA test, the *t-*test was used to determine pairwise differences between means. All statistical analyses were carried out by using SPSS (IBM SPSS 23.0, SPSS Inc.).

## Results

### Nutritional components of cow milk powder, goat milk powder, and sheep milk powder

The macronutrient analysis revealed sheep milk powder as the richest one in protein and fat, cow milk powder as the highest one in lactose, and goat milk powder was leading in carbohydrates (Table 1).

**Table 1:** Moisture and ash content of cow milk powder, goat milk powder, and sheep milk powder (g/100 g).

Components	Cow milk powder	Goat milk powder	Sheep milk powder
Protein	25.35 ± 0.03	25.05 ± 0.45	30.20 ± 2.70
Fat	29.25 ± 1.90	28.70 ± 1.50	30.80 ± 1.70
Lactose	37.10 ± 2.53	31.8 ± 1.00	23.25 ± 3.25
Carbohydrates	36.40 ± 1.60	36.70 ± 0.40	30.35 ± 4.65
Ash	5.25 ± 0.10	6.15 ± 0.65	5.30 ± 0.20
Moisture	3.75 ± 0.17	3.40 ± 0.02	3.35 ± 0.05

The differences between groups in the table did not reach statistical significance (*p* < 0.05) and are therefore not indicated.

### Fatty acid of cow milk powder, goat milk powder, and sheep milk powder

The fatty acid analysis of cow, goat, and sheep milk powders (Tables 2, 3) showed significant differences. Goat milk powder had the highest total fatty acid content (30.54 g/100 g), followed by sheep milk powder (29.60 g/100 g), and cow milk powder (27.03 g/100 g). Sheep milk powder exhibited the highest levels of monounsaturated and polyunsaturated fatty acids, *Ω*-3, arachidonic acid, and *α*-linolenic acid among the three types. Cow milk powder had the lowest content of saturated, monounsaturated, and polyunsaturated fatty acids.

**Table 2:** Fatty acid of cow milk powder, goat milk powder, and sheep milk powder (g/100 g).

Fatty acid	Cow milk powder		Goat milk powder		Sheep milk powder	
	Content	Ratio	Content	Ratio	Content	Ratio
C4:0	0.27 ± 0.04	0.99%	0.23 ± 0.02	0.75%	0.30 ± 0.09	1.01%
C6:0	0.26 ± 0.06	0.98%	0.33 ± 0.00	1.08%	0.28 ± 0.10	0.94%
C8:0	0.22 ± 0.08^a^	0.82%	0.48 ± 0.01^b^	1.59%	0.32 ± 0.13^ab^	1.09%
C10:0	0.70 ± 0.24^a^	2.60%	2.15 ± 0.03^b^	7.03%	1.25 ± 0.51^ab^	4.23%
C12:0	1.11 ± 0.06	4.12%	1.23 ± 0.01	4.04%	0.92 ± 0.32	3.12%
C13:0	0.06 ± 0.00^a^	0.23%	0.05 ± 0.00^ab^	0.15%	0.04 ± 0.01^b^	0.13%
C14:0	3.53 ± 0.05	13.07%	3.09 ± 0.10	10.13%	3.22 ± 0.80	10.87%
C14:1	0.28 ± 0.00	1.03%	0.04 ± 0.06	0.14%	0.06 ± 0.03	0.20%
C15:0	0.39 ± 0.04	1.44%	0.31 ± 0.01	1.03%	0.40 ± 0.08	1.35%
C16:0	9.55 ± 0.80	35.35%	9.39 ± 1.21	30.76%	8.90 ± 2.42	30.06%
C16:1	0.41 ± 0.02	1.52%	0.19 ± 0.09	0.61%	0.28 ± 0.13	0.93%
C17:0	0.18 ± 0.03	0.65%	0.27 ± 0.06	0.89%	0.32 ± 0.07	1.08%
C17:1	0.06 ± 0.01	0.21%	0.07 ± 0.00	0.22%	0.09 ± 0.03	0.30%
C18:0	3.05 ± 0.46	11.28%	3.79 ± 0.04	12.42%	3.79 ± 0.84	12.82%
C18:1n9t	0.63 ± 0.55	2.33%	0.71 ± 0.41	2.34%	0.71 ± 0.40	2.39%
C18:1n9c	5.28 ± 0.15	19.54%	6.68 ± 0.53	21.89%	6.97 ± 1.52	23.54%
C18:2n6t	0.04 ± 0.04	0.13%	0.04 ± 0.02	0.13%	0.05 ± 0.05	0.16%
C18:2n6c	0.50 ± 0.17	1.84%	0.94 ± 0.18	3.09%	0.81 ± 0.29	2.75%
C20:0	0.04 ± 0.01	0.15%	0.08 ± 0.01	0.27%	0.13 ± 0.05	0.43%
C18:3n6	0.00 ± 0.00	0.00%	0.00 ± 0.00	0.00%	0.01 ± 0.01	0.03%
C20:1	0.02 ± 0.01	0.09%	0.02 ± 0.01	0.06%	0.02 ± 0.01	0.06%
C18:3n3	0.18 ± 0.08	0.67%	0.16 ± 0.03	0.54%	0.27 ± 0.10	0.91%
C21:0	0.20 ± 0.05	0.72%	0.18 ± 0.09	0.58%	0.24 ± 0.10	0.80%
C22:0	0.00 ± 0.00	0.00%	0.02 ± 0.00	0.06%	0.05 ± 0.03	0.16%
C22:1n9	0.00 ± 0.00	0.00%	0.00 ± 0.00	0.00%	0.01 ± 0.01	0.02%
C20:4n6	0.01 ± 0.01	0.05%	0.05 ± 0.01	0.17%	0.06 ± 0.03	0.20%
C23:0	0.00 ± 0.01	0.00%	0.00 ± 0.00	0.01%	0.03 ± 0.01	0.10%
C22:2	0.02 ± 0.01	0.07%	0.00 ± 0.01	0.01%	0.02 ± 0.02	0.08%
C24:0	0.01 ± 0.01	0.04%	0.00 ± 0.00	0.01%	0.03 ± 0.01	0.10%
C20:5n3	0.02 ± 0.01	0.08%	0.01 ± 0.01	0.02%	0.03 ± 0.01	0.09%
C24:1	0.00 ± 0.00	0.00%	0.00 ± 0.00	0.00%	0.01 ± 0.01	0.02%
C22:6n3	0.00 ± 0.00	0.00%	0.00 ± 0.00	0.00%	0.01 ± 0.01	0.04%
Total	27.03 ± 1.53		30.54 ± 2.19		29.60 ± 6.34	

The differences between groups in the table did not reach statistical significance (*P* < 0.05) and are therefore not indicated.

^a^Indicated statistically significant differences compared with goat milk powder (*p* < 0.05).

^b^Indicated statistically significant differences compared with cow milk powder (*p* < 0.05).

**Table 3:** The categorized fatty acid content (g/100 g) and its proportion relative to the total fatty acids in three types of powdered milk.

Categorized fatty acid	Cow milk powder		Goat milk powder		Sheep milk powder	
	Content	Ratio	Content	Ratio	Content	Ratio
SFA	19.58 ± 1.12	72.44%	21.62 ± 1.50	70.79%	20.21 ± 4.60	68.29%
MUFA	6.68 ± 0.28	24.72%	7.71 ± 0.60	25.26%	8.13 ± 1.53	27.46%
PUFA	0.77 ± 0.13	2.84%	1.21 ± 0.15	3.96%	1.26 ± 0.26	4.26%
Ω-6	0.57 ± 0.22	73.64%	1.04 ± 0.23	85.84%	0.95 ± 0.30	75.40%
Ω-3	0.20 ± 0.04	26.36%	0.17 ± 0.09	14.16%	0.31 ± 0.12	24.60%
Arachidonic acid	0.01 ± 0.01	0.05%	0.05 ± 0.01	0.17%	0.06 ± 0.03	0.20%
Linoleic acid	0.49 ± 0.16	1.81%	0.98 ± 0.21	3.22%	0.86 ± 0.27	2.90%
*α*-linolenic acid	0.18 ± 0.03	0.67%	0.16 ± 0.08	0.54%	0.27 ± 0.10	0.91%
Total fatty acid	27.03 ± 1.53		30.54 ± 2.19		29.60 ± 6.34	

The differences between groups in the table did not reach statistical significance (*P* < 0.05) and are therefore not indicated.

### Protein nutritional composition analysis of three types of powdered milk

Sheep milk powder showed the highest total amino acid content (25.07 g/100 g) among cow, goat, and sheep milk powders (Tables 4, 5). It also had the highest proportions of individual amino acids to total proteins, reaching 83.0%. Cow and goat milk powders exhibited notable levels of specific amino acids like glutamic acid, leucine, and lysine, but overall, sheep milk powder outperformed in amino acid composition.

**Table 4:** The amino acid content (g/100 g) of three types of powdered milk.

Amino acid	Cow milk powder	Goat milk powder	Sheep milk powder
ASP	1.39 ± 0.37	1.18 ± 0.38	1.85 ± 0.74
THR	0.79 ± 0.22	0.82 ± 0.26	1.04 ± 0.42
SER	1.01 ± 0.27	0.87 ± 0.28	1.27 ± 0.49
GLU	3.89 ± 1.05	3.36 ± 1.03	4.93 ± 1.89
GLY	0.34 ± 0.10	0.29 ± 0.10	0.46 ± 0.19
ALA	0.60 ± 0.17	0.52 ± 0.18	0.90 ± 0.37
VAL	1.12 ± 0.34	1.10 ± 0.35	1.54 ± 0.62
MET	0.49 ± 0.14	0.42 ± 0.14	0.66 ± 0.26
ILE	0.92 ± 0.28	0.78 ± 0.25	1.18 ± 0.47
LEU	1.96 ± 0.54	1.78 ± 0.57	2.71 ± 1.07
TYR	0.89 ± 0.26	0.68 ± 0.25	1.11 ± 0.46
PHE	0.88 ± 0.26	0.80 ± 0.27	1.14 ± 0.46
LYS	1.49 ± 0.43	1.32 ± 0.43	1.96 ± 0.75
HIS	0.49 ± 0.15	0.44 ± 0.14	0.64 ± 0.26
ARG	0.62 ± 0.19	0.49 ± 0.17	0.79 ± 0.33
PRO	1.76 ± 0.51	1.70 ± 0.52	2.38 ± 0.92
TRP	0.23 ± 0.03	0.21 ± 0.01	0.27 ± 0.00
CYS	0.21 ± 0.02	0.23 ± 0.01	0.25 ± 0.02
EAA	8.37 ± 0.51	7.67 ± 0.46	11.12 ± 0.70
NEAA	9.62 ± 1.12	8.41 ± 0.99	12.58 ± 1.1.42
CEAA	1.09 ± 0.12	0.91 ± 0.12	1.36 ± 0.22
TAA	19.08 ± 5.22	16.99 ± 5.11	25.07 ± 8.46
EAA/TAA (%)	43.87	45.14	44.36
EAA/NEAA (%)	87.00	91.20	88.39

The differences between groups in the table did not reach statistical significance (*P* < 0.05) and are therefore not indicated. ASP, aspartic acid; THR, threonine; SER, serine; GLU, glutamic acid; GLY, glycine; ALA, alanine; VAL, valine; MET, methionine; ILE, isoleucine; LEU, leucine; TYR, tyrosine; PHE, phenylalanine; LYS, lysine; HIS, histidine; ARG, arginine; PRO, proline; TRP, tryptophan; CYS, cysteine; EAA, essential amino acids; NEAA, non-essential amino acids; CEAA, conditionally essential amino acids; TAA, total amino acids.

**Table 5:** The proportion of amino acids to total amino acids and total proteins in three types of powdered milk.

	The proportion of amino acids to total amino acids.			The proportion of amino acids to total proteins.		
	Cow milk powder	Goat milk powder	Sheep milk powder	Cow milk powder	Goat milk powder	Sheep milk powder
ASP	7.28%	6.94%	7.38%	5.48%	4.71%	6.13%
THR	4.14%	4.80%	4.15%	3.11%	3.26%	3.45%
SER	5.29%	5.10%	5.07%	3.98%	3.46%	4.21%
GLU	20.39%	19.79%	19.66%	15.34%	13.42%	16.32%
GLY	1.78%	1.70%	1.84%	1.34%	1.15%	1.52%
ALA	3.14%	3.06%	3.57%	2.36%	2.07%	2.97%
VAL	5.89%	6.47%	6.13%	4.43%	4.39%	5.09%
MET	2.58%	2.49%	2.62%	1.94%	1.69%	2.17%
ILE	4.82%	4.60%	4.69%	3.63%	3.12%	3.89%
LEU	10.29%	10.47%	10.82%	7.74%	7.10%	8.98%
TYR	4.65%	4.01%	4.44%	3.50%	2.72%	3.68%
PHE	4.62%	4.71%	4.54%	3.48%	3.20%	3.77%
LYS	7.80%	7.77%	7.80%	5.87%	5.27%	6.47%
HIS	2.57%	2.60%	2.55%	1.93%	1.76%	2.12%
ARG	3.27%	2.87%	3.16%	2.46%	1.95%	2.62%
PRO	9.24%	10.03%	9.48%	6.96%	6.80%	7.87%
TRP	1.20%	1.25%	1.06%	0.90%	0.85%	0.88%
CYS	1.08%	1.37%	1.00%	0.81%	0.93%	0.83%
TAA				75.28%	67.84%	83.00%

The differences between groups in the table did not reach statistical significance (*P* < 0.05) and are therefore not indicated. ASP, aspartic acid; THR, threonine; SER, serine; GLU, glutamic acid; GLY, glycine; ALA, alanine; VAL, valine; MET, methionine; ILE, isoleucine; LEU, leucine; TYR, tyrosine; PHE, phenylalanine; LYS, lysine; HIS, histidine; ARG, arginine; PRO, proline; TRP, tryptophan; CYS, cysteine; TAA, total amino acids.

### Analysis of the vitamin composition of three types of powdered milk

Sheep milk powder contained the highest levels of vitamin A, vitamin B1, and vitamin B2 among cow, goat, and sheep milk powders (Table 6). Cow milk powder had the highest folate content, while goat milk powder showed the lowest levels of vitamin B1 and B2.

**Table 6:** Analysis of the vitamin composition of three types of powdered milk.

Vitamin	Cow milk powder	Goat milk powder	Sheep milk powder
vitamin A (μg/100 g)	270.00 ± 12.00	300.50 ± 54.50	355.50 ± 113.50
vitamin B_1_ (mg/100 g)	0.14 ± 0.022	0.084 ± 0.018	0.24 ± 0.0055
vitamin B_2_ (mg/100 g)	1.22 ± 0.25	0.69 ± 0.12	1.70 ± 0.28
Folic acid (μg/100 g)	59.05 ± 6.05	50.90 ± 6.60	57.20 ± 10.90

The differences between groups in the table did not reach statistical significance (*P* < 0.05) and are therefore not indicated.

### Mineral element analysis of three types of powdered milk

Sheep milk powder showed the highest calcium content among cow, goat, and sheep milk powders, while goat milk powder exceled in potassium and magnesium levels (Table 7). Cow milk powder generally exhibited lower mineral levels compared to the other types, except for sodium and zinc, where it surpassed both goat and sheep milk powders.

**Table 7:** Analysis of the mineral element composition of three types of powdered milk.

Mineral element	Cow milk powder	Goat milk powder	Sheep milk powder
Calcium (g/kg)	8.67 ± 0.52	9.57 ± 0.07	10.70 ± 0.20
Potassium (g/kg)	11.05 ± 0.35	14.30 ± 3.50	8.35 ± 0.79
Magnesium (mg/kg)	708.50 ± 48.50	920.50 ± 109.50	897.00 ± 6.00
Sodium (g/kg)	2.83 ± 0.33	2.75 ± 0.23	2.75 ± 0.39
Zinc (mg/kg)	26.30 ± 1.90	24.35 ± 1.45	26.25 ± 0.85

The differences between groups in the table did not reach statistical significance (*P* < 0.05) and are therefore not indicated.

### The growth status of rats fed three types of powdered milk

**Figure 1:**
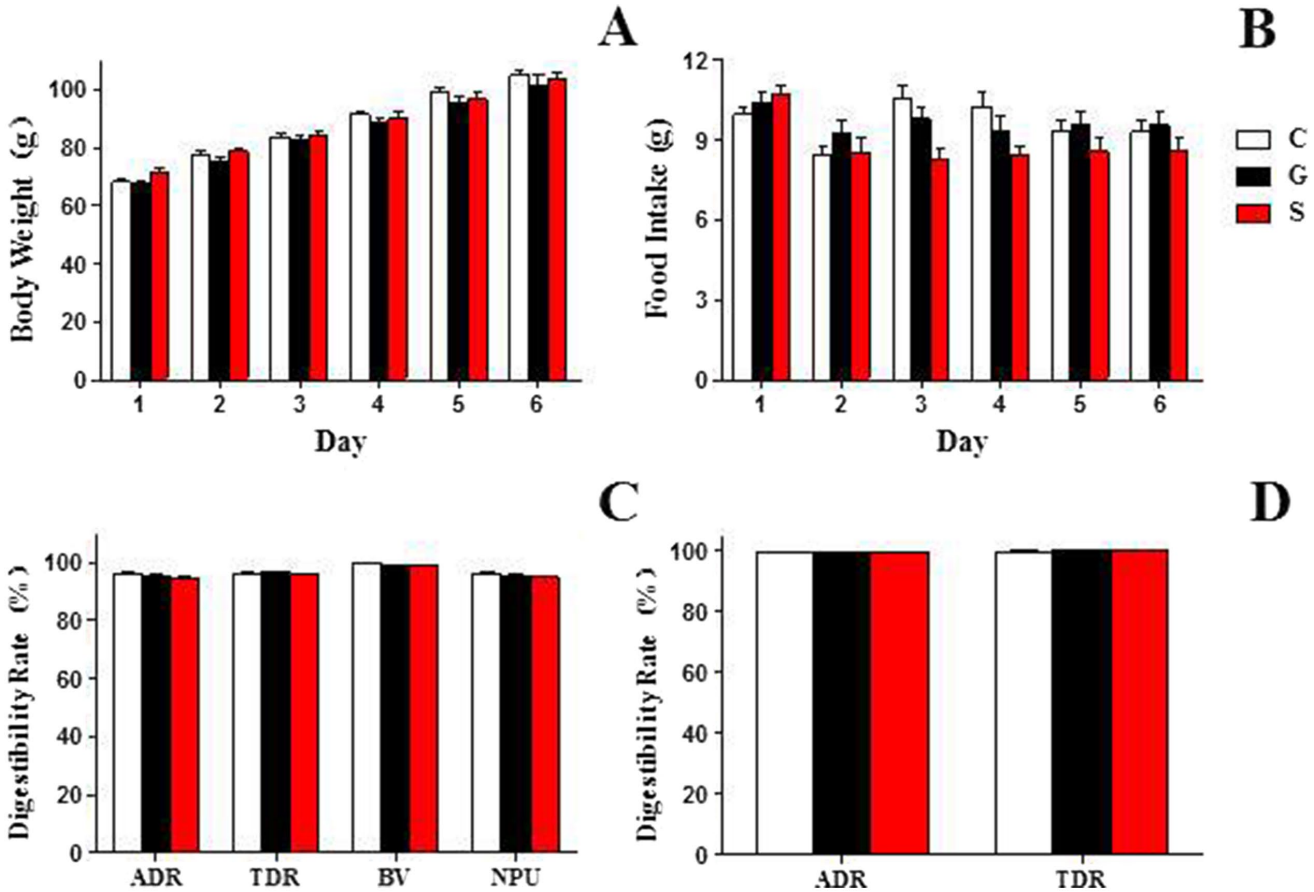
The growth status and digestion and absorption profiles of rats fed three types of powdered milk feed. (A) The body weight changes among rat groups consuming the three types of powdered milk feed. (B) The food intake among rat groups fed with the three types of powdered milk feed. (C) The digestibility and absorption rates of protein from the three different types of powdered milk feed. (D) The digestibility rates of fat from the three different types of powdered milk feed. ADR, apparent digestibility rate; TDR, true digestibility rate; BV, biological value; NPU, net protein utilization rate. C, Cow milk powder group (*n* = 20); G, Goat milk powder group (*n* = 20); S, Sheep milk powder group (*n* = 20). The differences between groups in the table did not reach statistical significance (*p* < 0.05) and are therefore not indicated.

The changes in rat body weight after 7 days of feeding with three different types of milk powder were depicted in Figures 1A,B. There were minimal differences in food intake among the rat groups fed with the three types of milk powder, however, a consistent trend was observed across all three milk powder groups, with rat body weight showing an increasing tendency.

### The digestion and absorption profiles of three types of powdered milk

This study investigated the digestibility and absorption rates of three types of milk powder proteins (Figure 1C): goat, sheep, and cow milk powders. Digestibility rates (%) for goat, sheep, and cow milk powders were 95.39 ± 0.44, 94.80 ± 0.15, and 95.19 ± 1.11, respectively. True digestibility (%) values were 96.54 ± 0.38, 95.99 ± 0.27, and 96.34 ± 0.82, respectively. Biological values (%) were 98.93 ± 0.37, 98.95 ± 0.30, and 99.86 ± 0.18, and net utilization rates (%) were 95.50 ± 0.71, 94.98 ± 0.47, and 96.20 ± 0.92 for goat, sheep, and cow milk powders, respectively. For milk powder fat (Figure 1D), digestibility rates (%) were 99.52 ± 0.06, 99.61 ± 0.10, and 99.39 ± 0.15, and true digestibility (%) values were 99.96 ± 0.17, 100.07 ± 0.08, and 99.83 ± 0.19 for goat, sheep, and cow milk powders, respectively.

### Metabolite content analysis of three types of powdered milk group

**Figure 2:**
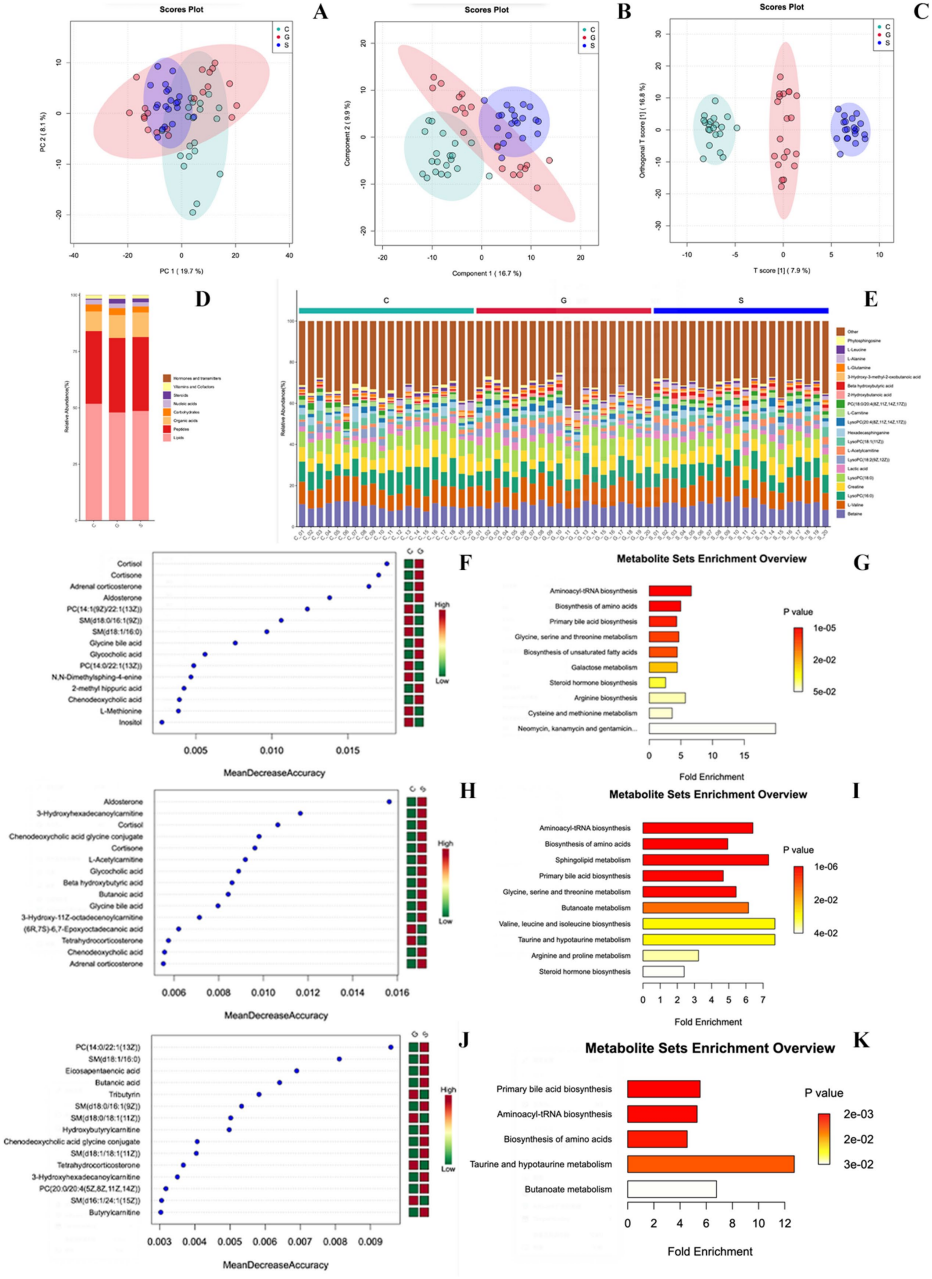
Metabolomics analysis of three powdered milk groups. (A) PCA of metabolites. (B) PLS-DA of metabolites. (C) O-PLS-DA of metabolites. (D) Biomolecule categories among groups (Lipids, Peptides, Organic acids, Carbohydrates, Nucleic acids, Steroids, Vitamins, Cofactors, Hormones, and transmitters). (E) Different metabolites per sample. (F) Random forest algorithm for differential metabolites (Cow vs. Goat). (G) Over-Representation Analysis (ORA) based on KEGG pathways (Cow vs. Goat). (H) Random forest algorithm for differential metabolites (Cow vs. Sheep). (I) ORA based on KEGG pathways (Cow vs. Sheep). (J) Random forest algorithm for differential metabolites (Goat vs. Sheep). (K) ORA based on KEGG pathways (Goat vs. Sheep). C, Cow milk powder (*n* = 20); G, Goat milk powder (*n* = 20); S, Sheep milk powder (*n* = 20).

Metabolite analysis of three types of powdered milk showed minimal differences in overall metabolic composition based on PCA (Figure 2A). Further analysis by using PLS-DA and O-PLS-DA (16) revealed distinct metabolite profiles among cow, sheep, and goat milk powders (Figures 2B,C). Cow milk powder was notably higher in lipids, sheep milk powder in organic acids, and goat milk powder in steroids. Peptide and nucleic acid contents were relatively consistent across all groups, while vitamins, cofactors, hormones, and transmitters showed minimal variation or negligible presence (Figures 2D,E).

### Metabolite identification

The study indicates that the percentage composition of metabolites in serum samples from three types of powdered milk has been identified and quantified. This analysis is visually represented by using stacked bar charts, facilitating direct comparisons of metabolic differences between groups. Figures 2F–K highlights the top 20 metabolites by abundance, grouping the remaining under ‘Others’. The random forest algorithm was applied to identify differential metabolites between different three types of powdered milk groups (Figures 2F,H,J). Over-Representation Analysis (ORA) based on KEGG pathways elucidated key biological pathways influencing metabolic processes in the serum of rats under different formula-fed regimens (Figures 2G,I,K).

Comparative analysis between cow milk powder and goat milk powder groups (Figures 2F,G) revealed significant differences, particularly in metabolites related to steroid hormone biosynthesis (e.g., cortisol and aldosterone) and bile acid metabolism. Similar comparisons between cow milk powder and sheep milk powder groups (Figures 2H,I) highlighted associations with hormonal regulation and pathways such as amino acid and lipid metabolism. Comparing sheep milk powder and goat milk powder groups (Figures 2J,K) uncovered significant associations with various metabolic pathways, including lipid metabolism and amino acid synthesis, influenced by specific metabolites like primary bile acids and fatty acids.

## Discussion

The study provides a comprehensive comparative analysis of the nutritional composition, growth performance, digestibility, and metabolomic profiles of milk powder diets from cows, goats, and sheep in rat models. The comprehensive comparative analysis of milk powders from sheep, goats, and cows reveals significant variations in both macronutrient and micronutrient compositions. Sheep milk powder stands out with the highest protein content and lowest lactose content among the three types, while goat milk powder shows the highest carbohydrate content. Additionally, sheep milk powder demonstrates superior levels of monounsaturated and polyunsaturated fatty acids, including essential fatty acids such as *Ω*-3, arachidonic acid, and *α*-linolenic acid, distinguishing it from the other types. Sheep milk powder also exhibits the highest total amino acid content and proportion to total proteins, with notable elevations in specific amino acids like glutamic acid, leucine, proline, lysine, aspartic acid, valine, and serine, highlighting its nutritional superiority.

Previous studies have demonstrated that sheep colostrum typically contains higher protein levels compared to other domesticated animal milks, including water buffalo (17, 18), camel (19), cattle (20), goat (21), horse (22), and donkey colostrum (23). Previous research has also observed that sheep milk had a higher total fat content compared to cow (24) or goat milk (25). Cow’s milk generally has a higher lactose content compared to goat and sheep milk, which makes it harder to digest (26). Sheep milk powder stood out with the lowest carbohydrate content, for those needing to control carbohydrate intake, sheep milk powder may be a preferable option. Sheep milk powder contains the highest levels of monounsaturated fatty acids, polyunsaturated fatty acids, omega-3 fatty acids, arachidonic acid, and alpha-linolenic acid among the three types of powdered milk. These findings are consistent with previous research (24), indicating that sheep milk tends to exhibit a higher proportion of omega-3 polyunsaturated fatty acids (PUFAs) and PUFA bio hydrogenation intermediates (PUFA-BHIs), such as conjugated linoleic acid (CLA), trans-mono-unsaturated fatty acids (t-MUFAs), vaccenic acid (VA), rumenic acid (RA), and branched-chain fatty acids (BCFAs), compared to cow milk. This difference is attributed to a less extensive bio hydrogenation process in sheep milk (24). This study confirms that sheep milk powder exhibits a markedly higher total amino acid content and proportion relative to total proteins compared to both cow and goat milk powders.

This finding is consistent with previous research (27) and is underscored by notable increases in specific amino acids, namely glutamic acid, leucine, proline, lysine, aspartic acid, valine, and serine. Furthermore, the amino acid profiles derived from milks power of cows, sheep, and goats display distinct characteristics, serving as hallmark indicators of these respective species.

The analysis of three types of powdered milk unveils significant disparities in both vitamin and mineral compositions, with sheep milk powder exhibiting the highest levels of vitamin A, B1, and B2, along with superior calcium content, while cow milk powder excels in folate content and demonstrates higher sodium and zinc levels, and goat milk powder presents the lowest levels of vitamin B1 and B2, as well as distinctive potassium and magnesium levels. The variation in the concentration of different components found in milk depends on mammalian species, genetic, physiological, nutritional factors, and environmental conditions, concentrations of different vitamin and mineral components vary depending on mammalian species, genetics, physiological factors, nutritional requirements, and environmental factors (28).

The study investigates the growth performance and digestion kinetics of rats fed with three types of powdered milk. Despite minimal differences in food intake, all three milk powder groups exhibited a consistent trend of increased body weight over 7 days. Assessment of protein and fat digestibility revealed high apparent and true digestibility rates across goat, sheep, and cow milk powder groups, with slight variations evaluated, demonstrating high apparent and true digestibility rates with slight variations among the groups. This study also found that the protein digestibility revealed high rates across goat, sheep, and cow milk powder groups, surpassing previous researches (29–31). The mean digestibility of goat milk protein (94.0%) was similar to that of cow milk protein isolate and skimmed milk protein (95.1 and 95.5%) (32, 33). Additionally, it compared reasonably well (94.0%) with the digestibility of goat milk protein concentrate in rats (34). The high digestibility of three type of milk protein renders it a crucial animal-sourced foodstuff for populations predominantly adhering to vegetarian diets (35) and in countries where meat consumption frequency is low, mitigating the risk of inadequate quality protein intake (36). From both nutritional and economic perspectives, fat is one of the most critical components of milk. This research has also shown that the true digestibility rate for fat content in the three types of milk powders is approximately 99%. The study indicates that milk fat intake is often associated with a higher risk of cardiovascular disease (CVD) due to its significant saturated fat content. Yet, it is important to understand that saturated fatty acids (SFAs) are not a monolithic group; they have structural differences that can lead to varying impacts on biological processes (37). Therefore, future research should pay attention to the impact of the structure of milk fatty acids on health.

In spite of the worldwide consumption of bovine milk, small ruminant milk, such as goat’s and sheep’s milk, is gaining a great deal of attention, especially in the Mediterranean region (38). This study analyzed serum metabolites in rats fed with cow, goat, and sheep milk powders, identifying key differences through random forest analysis and pathway enrichment. Comparing the cow, goat, and sheep milk powder groups revealed distinct metabolic signatures *in vivo*. In comparison to the cow milk powder group, metabolites in the serum of rats fed goat milk powder, such as cortisol, cortisone, adrenal corticosterone, and aldosterone, which are essential for steroid hormone biosynthesis, highlight the significance of this pathway in the synthesis of crucial adrenal cortex hormones. Simultaneously, glycine bile acid and glycocholic acid play a crucial role in the metabolism of glycine, serine, and threonine, connecting to primary bile acid biosynthesis through the generation and metabolism of bile acids. Metabolites in the serum of rats fed sheep milk powder, including hormonal regulators such as aldosterone, cortisol, and tetrahydrocorticosterone, as well as bile acids like chenodeoxycholic acid glycine conjugate, actively engage in bile acid pathways while being modulated by taurine and hypotaurine metabolism. Due to their close phylogenetic relationship, the goat milk powder-fed group and the sheep milk powder-fed group exhibit fewer enriched metabolic pathways *in vivo* compared to the milk powder-fed group derived from species such as cows. These pathways mainly involve lipid metabolism and its regulation by specific metabolites, notably within butanoate metabolism and primary bile acid biosynthesis. Within butanoate metabolism, metabolites such as eicosapentaenoic acid, butanoic acid, and tributyrin serve as regulators, impacting the metabolism of short-chain fatty acids. Steroid hormones play an essential role in regulating water and salt balance, metabolism, and stress response, as well as in initiating and maintaining sexual differentiation and reproduction (39). Bile acids facilitate the absorption of lipids in the gut but are also involved in maintaining cholesterol homeostasis, releasing bile, excreting toxic substances, and controlling energy metabolism. The synthesis of bile acids, including cholic acid and chenodeoxycholic acid, is a complex process involving at least 17 enzymes and metabolite transport proteins. Disorders of bile acid synthesis can affect individuals in various ways, ranging from cholestatic liver disease to neuropsychiatric symptoms and spastic paraplegia (40). Butanoate metabolism is the metabolic fate of several short-chain fatty acids or short-chain alcohols typically produced by intestinal fermentation. These molecules are eventually converted into ketone bodies, short-chain lipids, citrate cycle precursors, glycolysis precursors, or glutamine precursors (41). Overall, this study provides comprehensive insights into the metabolic profiles and pathway associations influenced by different formula-fed regimens in rat serum samples.

## Conclusion

In summary, this study reveals significant disparities in nutritional composition and metabolic responses among cow, goat, and sheep milk powders. Sheep milk powder demonstrates a superior nutritional profile, with elevated levels of protein, essential fatty acids, amino acids, vitamins, and minerals. All three milk powders exhibit high rates of protein and fat digestibility, highlighting their efficacy as quality nutrition sources for diverse dietary needs. Furthermore, distinct metabolic signatures were identified in rats administered these milk powder formulations, emphasizing the crucial roles of steroid hormone biosynthesis and bile acid metabolism in eliciting physiological responses. Notably, despite their close evolutionary relationship, goat and sheep milk powders display unique metabolic enrichments, especially in lipid metabolism pathways. This study offers valuable insights into the metabolic implications of different milk powder sources, informing dietary choices and facilitating the development of targeted public health strategies to optimize nutritional intake and promote overall well-being.

## Data Availability

The raw data supporting the conclusions of this article will be made available by the authors, without undue reservation.

## Footnotes

^1^https://www.bioincloud.tech
