# Supplementary material for: Comparative analysis of the nutritional composition, digestibility, metabolomics profiles and growth influence of cow, goat and sheep milk powder diets in rat models
Source: Front Nutr. 2024 Nov 22;11:1428938. doi: 10.3389/fnut.2024.1428938 (PMC11622695; doi:10.3389/fnut.2024.1428938)
Supplement: Supplementary file 1 [file Table_1.DOCX]

**Supplementary**

Table .S1. The formulation of cow milk powder feed , goat milk powder feed , and sheep milk powder feed (g/100g).

| Components | Cow milk powder feed | Goat milk powder feed | Sheep milk powder feed |
| --- | --- | --- | --- |
| Protein | 28.64±0.57 | 28.73±0.56 | 28.56±0.86 |
| Fat | 29.17±0.10 | 29.40±0.42 | 29.18±0.06 |
| Lactose | 37.10±2.53 | 31.8±1.00 | 23.25±3.25 |
| Carbohydrates | 36.40±1.60 | 36.70±0.40 | 30.35±4.65 |
| Ash | 5.25±0.10 | 6.15±0.65 | 5.30±0.20 |
| Moisture | 3.75±0.17 | 3.40±0.02 | 3.35±0.05 |

Table.S2.The formulation for the N-free diet (g/100g).

| Components | N-free diet |
| --- | --- |
| fat | 27.0 |
| saccharose | 27.8 |
| cellulose | 10.0 |
| maltodextrin | 27.0 |
| moisture | 3.5 |
| vitamins (including choline） | 4.7  according to AIN93 standards |
| minerals |  |
| total | 100 |
